# Self‐reported physical activity more than 1 year after stroke and its determinants in relation to the WHO recommendations

**DOI:** 10.1002/pmrj.13297

**Published:** 2025-01-03

**Authors:** Maria Kähler, Hanna M. Nilsson, Lina Rosengren, Lars Jacobsson, Jan Lexell

**Affiliations:** ^1^ Department of Health Sciences, Rehabilitation Medicine Research Group Lund University Lund Sweden; ^2^ Department of Rehabilitation Sunderby Hospital Luleå Sweden; ^3^ Department of Rehabilitation Ängelholm Hospital Ängelholm Sweden; ^4^ Department of Health, Education and Technology Luleå University of Technology Luleå Sweden

## Abstract

**Background:**

Physical activity (PA) after stroke has significant health benefits if it is conducted regularly, with sufficient intensity and duration. Because of the health benefits, it is important to identify those below the World Health Organization (WHO) recommended level of PA. However, few studies have assessed the level of PA after stroke in relation to the WHO recommendations and which sociodemographic factors and stroke characteristics are associated with those below the WHO recommendations.

**Objective:**

To assess survivors of stroke at least 1 year after onset and (1) describe their self‐reported level of PA; (2) explore the association between PA, sociodemographics, and stroke characteristics, and (3) determine the characteristics of those below the WHO recommended level of PA.

**Design:**

Cross‐sectional descriptive survey.

**Setting:**

Community settings.

**Participants:**

Data were collected from 160 survivors of stroke (mean age 73 years, 46% women, mean time since stroke onset 35 months).

**Interventions:**

Not applicable.

**Main Outcome Measures:**

The Swedish National Board of Health and Welfare Physical Activity Questionnaire and the following sociodemographics and stroke characteristics: gender, age, marital status, vocational situation, need for home help, use of mobility devices, time since stroke onset, first‐time stroke, type of stroke, location of stroke, and stroke treatment.

**Results:**

Two thirds (66.3%) of the participants were below the WHO recommendations. The hierarchical regression analysis explained 13% of the variance in PA with need for home help as a single significant contributor. Those who did not meet the WHO recommendations were significantly older, more likely to live alone, and in need of home help and mobility devices.

**Conclusions:**

A majority of survivors of stroke do not meet the WHO recommended level of PA. Future studies should assess how other factors characterize those who are physically inactive. This knowledge could help rehabilitation professionals to target interventions and self‐management programs to promote PA among survivors of stroke.

## INTRODUCTION

Stroke is the third leading cause of death and disability worldwide.^1^, ^2^ About 50% of survivors of stroke experience motor, sensory, and cognitive impairments.^3^ These impairments can affect the ability to be physically active.^4^, ^5^

Physical activity (PA), defined as any movement of the body that results in energy expenditure, can range from household chores to structured exercise.^6^ PA can be beneficial for survivors of stroke in several ways. For example, prestroke PA is associated with a less severe stroke^7^ and PA after stroke can improve recovery^8^ and reduce the risk of stroke recurrence.^9^ For PA to have significant health benefits it is important that it is conducted regularly and with sufficient intensity and duration.^10^

Studies have shown that people after stroke are less physically active than healthy controls, and their PA is low in both duration and intensity^11^, ^12^ and remains low a year after stroke onset.^13^ Men are reported to be somewhat more physically active than women after stroke,^14^ but studies also show no significant differences between men and women.^15^, ^16^ Those of higher age seem to be at greater risk of physical inactivity after stroke.^14^ Also, reduced walking ability and use of mobility devices were significantly associated with increased amount of sedentary time.^17^ A study where most of the participants were less than 1 year post stroke showed that living alone was significantly associated with performing moderate PA.^15^ Some previous studies have shown no associations between the level of PA and time since stroke onset and the location of stroke.^14^, ^15^ Knowledge about other stroke characteristics, such as type of stroke and treatment with thrombolysis and/or thrombectomy and their association with the level of PA, is still limited. In addition, studies on PA after stroke have mostly been performed within the first year after onset and our knowledge of the level of PA >1 year after stroke is also limited.

In 2020, the World Health Organization (WHO) published updated guidelines regarding PA and sedentary behavior, and, for the first time, people living with disabilities were included in the recommendations.^18^ The stroke population is recommended at least 150 minutes of moderate intensity or 75 minutes of vigorous intensity PA per week, which is the same recommendations as the adult (18 to >65 years) nondisabled population.^10^, ^19^ Because of the health benefits of PA after stroke, it is important to identify and determine the characteristics of those that do not meet the WHO recommendations.

Thus, the objectives of the present study are to assess survivors of stroke at least 1 year after onset and (1) describe their self‐reported level of PA; (2) explore the association between PA, sociodemographics and stroke characteristics; and (3) determine the characteristics of those below the WHO recommended level of PA.

## METHODS

### Study design

This is a cross‐sectional survey targeting adult survivors of stroke at least 1 year after stroke onset and is part of a larger study: the Life After Stroke In Northern Sweden Study (LASINS). LASINS has a combined quantitative exploratory and descriptive design with a rehabilitation medicine approach aiming to deepen our understanding of factors of importance for a successful and healthy life after stroke. Other studies within the LASINS aim to include factors such as sleep, depressive symptoms, fatigue, and quality of life. Complete information about the LASINS study with all the including assessment tools in the overall project, and their psychometric properties can be found in our previous study.^20^ In the present study a subset of the data from the LASINS is used (ie, sociodemographic data, stroke characteristics, and the participants' self‐reported level of PA). The Strengthening the Reporting of Observational Studies in Epidemiology guidelines (STROBE) are followed and the necessary items according to the STROBE checklist are included.^21^, ^22^

### Ethical considerations

All participants received information about the study, their voluntary participation, and the possibility to withdraw from the study at any time. Written informed consent was obtained before enrollment. The LASINS follows the principles of the Helsinki Declaration on research involving humans and has been approved by the Swedish Ethical Review Authority (April 25, 2021; 2021‐01408).

### Study participants

The included participants were admitted between January 2017 and December 2019 to the stroke unit at a regional hospital in Norrbotten County, the northernmost part of Sweden. We included people with a stroke diagnosis (*International Classification of Diseases, Tenth Revision*: I61 cerebral hemorrhage; I63 cerebral infarction; I64 acute cerebrovascular disease not specified as hemorrhage or infarction) who met the following criteria: over 18 years of age, living in their own home, and able to understand and answer questionnaires and self‐assessment tools in Swedish. We excluded people with a transient ischemic attack (TIA), dementia, or other severe cognitive impairments and those who had moved abroad.

Based on these criteria, we reviewed medical records to identify potential participants. A total of 1518 people had sustained a stroke and had been admitted to the hospital during the 3‐year period. Of these, 949 (63%) had been admitted to the stroke unit and 301 people met the inclusion criteria and were invited; the remaining people did not meet the inclusion criteria (*n* = 403; eg, TIA or living in a nursing home, severe cognitive impairment and dementia, not speaking/understanding Swedish, or moved abroad) or were deceased (*n* = 245). Finally, 160 people accepted to participate and comprise the final sample (response rate 53%).^20^

### Data collection

The potential participants (*n* = 301) were invited through mail between June 2021 and February 2022. They received written information about the study, an informed consent form, a questionnaire to collect sociodemographic data (ie, gender (man or woman), age, marital status, vocational situation, use of mobility device, and need of home help), the self‐assessment tools, and a prepaid envelope. One reminder was sent after the first invitation. Data about the participants' stroke characteristics (ie, time since stroke onset, type and location of stroke, stroke treatment [ie, thrombolysis and/or thrombectomy], if this was their first‐time stroke, and comorbidities) were obtained from the participants' medical records.

### Self‐reported level of physical activity

To assess the participants' level of PA, the Swedish National Board of Health and Welfare Physical Activity (BHW PA) questionnaire was used (see Appendix 1). The BHW PA questionnaire was designed by the Swedish National Board of Health and Welfare to assess the level of PA and identify those who do not meet the WHO recommendations of at least 150 minutes of moderate activity per week.^23^, ^24^ It comprises two questions about everyday PA and exercise. The categorical answers with seven (everyday PA) and six (exercise) possible responses are combined to a total PA score (2 × exercise + everyday PA) that ranges from 3 to 19; a greater score reflects a higher level of total PA. The cutoff score for meeting the recommendations of 150 minutes of moderate activity per week is 11; a score of ≤10 represents those who do not meet the WHO recommendations.^24^ The BHW PA questionnaire correlates with body mass index, VO_2_‐max, blood lipids and glucose, balance, and general health and is valid in comparison with objective methods such as accelerometer.^23^

### Statistical analysis

Data are presented using quantitative descriptive statistics with mean, median, SD, minimum and maximum, frequency, and proportion (%), where appropriate.

To explore the association between the participants' level of PA and sociodemographics and stroke characteristics, a hierarchical regression analysis was performed, with the level of PA as a dependent variable. As independent variables we included gender and age, and the following sociodemographics and stroke characteristics: marital status, vocational situation, need for home help, use of mobility devices, time since stroke, first‐time stroke, type of stroke, location of stroke, and stroke treatment. The variables “vocational situation,” “use of mobility devices,” and “type of stroke” had three or four response options and were transformed into bivariate variables. Dummy variables were created for “location of stroke” with left hemisphere as reference category and “cerebellum,” “brainstem,” and “other” were combined into a new category: “other.”

The hierarchical regression analysis was conducted in three steps: gender and age were included first, sociodemographics second, and finally stroke characteristics in the third step to assess if stroke characteristics added to the variance in PA. The largest variance inflation factor was 2.1 and no variable had a tolerance <0.48; therefore, there was no indication of multicollinearity. Also, a residual analysis was conducted to confirm the validity of the regression model.^25^

To determine the characteristics of those below the WHO recommended level of PA, the chi‐square and Mann–Whitney *U* tests were used, where appropriate. All statistical analyses were performed using the IBM SPSS Statistics Software v 28 (IBM Corporation, Armonk, NY, USA). *P* values < .05 were considered statistically significant.

## RESULTS

### Participant characteristics

The characteristics of the 160 participants are presented in Table 1. There were 86 men (54%) and 74 women (46%) with a mean age of 73 years (SD ±11; 30–91). The majority (*n* = 134; 84%) of the participants were not working and two thirds (*n* = 100; 62.5%) were cohabitant. A total of 134 participants (84%) did not need any home help and 113 participants (71%) did not use any mobility devices.

**TABLE 1:** Characteristics of the 160 participants.

Men	86 (54)
Women	74 (46)
Age (y)	73 ± 11; 74, 30–91
Marital status	
Living alone	60 (37.5)
Cohabitant	100 (62.5)
Vocational situation	
Working	26 (16)
Not working	134 (84)
Need for home help	26 (16)
Use of mobility devices^a^	47 (29)
Time since stroke onset (mo)	35 ± 11; 34, 18–61
First‐time stroke	136 (85)
Type of stroke	
Ischemia	139 (87)
Subarachnoid hemorrhage	0
Intracerebral hemorrhage	21 (13)
Location of stroke	
Right hemisphere	53 (33)
Left hemisphere	59 (37)
Cerebellum	13 (8)
Brainstem	3 (2)
Other^b^	32 (20)
Stroke treatment	
Thrombolysis	33 (21)
Thrombectomy	3 (2)
Comorbidity	143 (89)

*Note*: Data are presented as *n* (%) and mean ± SD; median, min‐max.

^a^ Use of mobility devices = walking aids and wheelchair.

^b^ Other = stroke localized in both hemispheres, multifocal stroke, or unidentified.

The mean time since stroke onset was 35 months (SD ±11, 18–61). Most participants had sustained an ischemic stroke (*n* = 139; 87%). A majority, 112 participants (70%), had a hemispheric stroke and the remainder a stroke located elsewhere in the brain. A total of 36 participants (23%) were treated with thrombolysis and/or thrombectomy. The majority had a first‐time stroke (*n* = 136; 85%) and some comorbidity (*n* = 143; 89%), where hypertension, atrial fibrillation, hyperlipidemia, and type 2 diabetes were most frequently reported.

### Self‐reported level of PA

**FIGURE 1:**
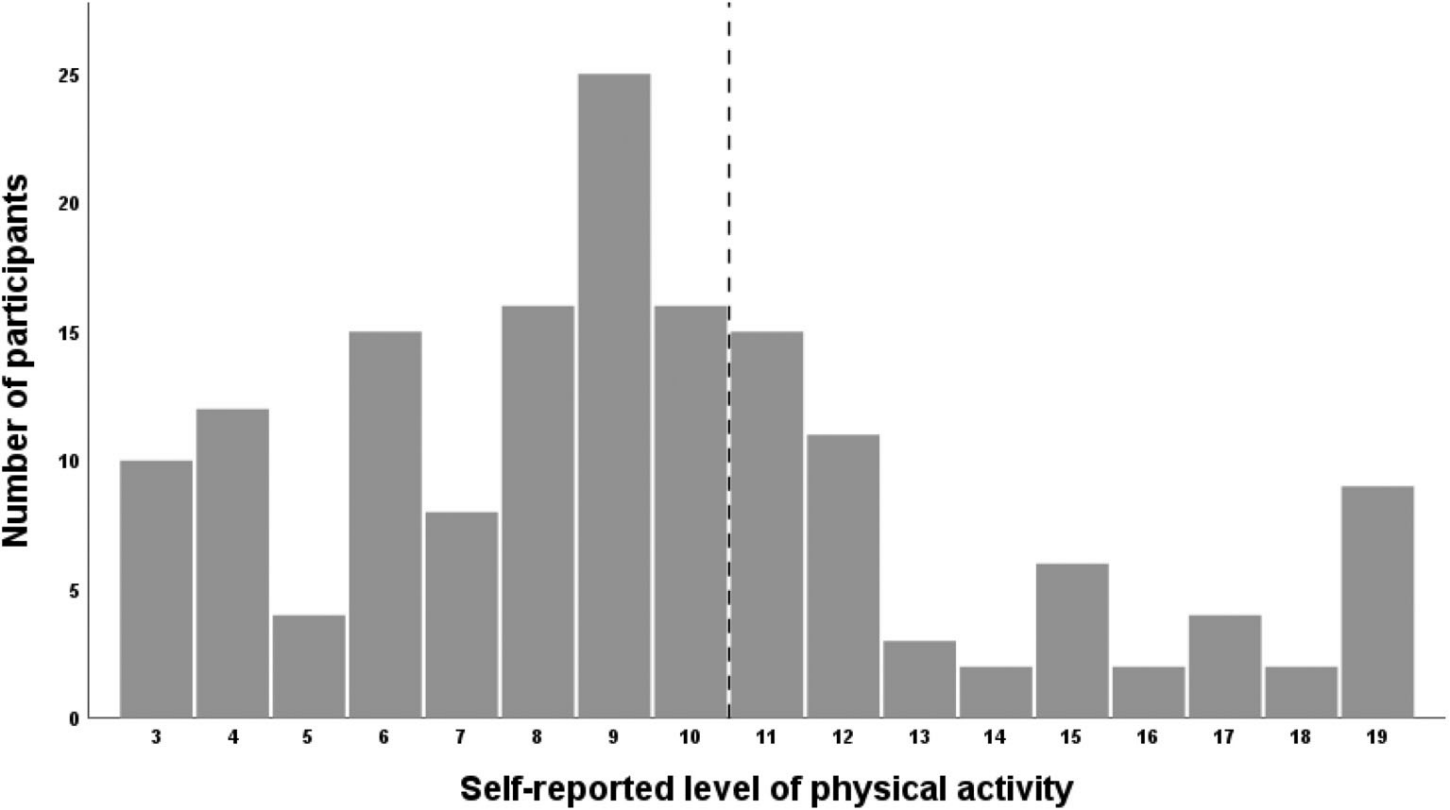
Self‐reported level of physical activity (PA) assessed with the Swedish National Board of Health and Welfare Physical Activity questionnaire. Two questions about PA are combined to a total PA score that ranges from 3 to 19, were a greater score reflects a higher level of PA. The vertical line represents the cutoff (ie, 11 points) between those who are below or above the World Health Organization recommended level of PA.

The participants' self‐reported level of PA is presented in Figure 1. It includes the whole range from 3 to 19, where a large proportion of the participants reported their level of PA just below the cutoff score of 11 (score 8–10; *n* = 57, 35.6%). The mean total PA score was 9.5 (SD ±4.2) and the median was 9. Two thirds (66.3%) of the participants had a score ≤10.

### Associations between the level of PA, sociodemographics, and stroke characteristics

The results of the hierarchical regression analysis with the level of PA as dependent variable are presented in Table 2. In the first step, where gender and age were included, age was a significant single contributor to the variance in the level of PA. In the second step, marital status, vocation, need for home help, and use of mobility devices were included. This resulted in a significant change in the model (Sig *F* change *p* < .001) and explained 13% (*R* ^2^ Adj 0.13; *p* < .001) of the variance in the level of PA with need for home help (*p* = .015) and use of mobility devices (*p* = .027) as significant contributors. In the third and final step, time since stroke, first‐time stroke, type and location of the stroke, and treatment (ie, thrombolysis and/or thrombectomy) were included in the model; this did not result in a significant change of the model. The final model explained 13% (*R* ^2^ Adj 0.13; *p* = .001) of the variance in the level of PA with need for home help as a single significant (*p* = .038) contributor.

**TABLE 2:** Results of the hierarchical regression analysis (3 steps) for the 160 participants with level of physical activity (PA) as dependent variable.

	Level of PA		
	Step 1	Step 2	Step 3
Gender	−0.04	0.00	0.01
Age	−0.22 **(.006)**	−0.00	−0.03
Marital status		0.04	0.00
Vocation		−0.05	−0.03
Need for home help		−0.23 **(.015)**	−0.20 **(.038)**
Use of mobility devices^a^		−0.20 **(.027)**	−0.17
Time since stroke			0.02
First time stroke			−0.10
Type of stroke			−0.04
Location of stroke^b^			
Right hemisphere			−0.04
Other^c^			0.05
Stroke treatment			0.13
Significance	.02	<.001	.001
*R* ^2^ adj	0.04	0.13	0.13
*R* ^2^ change	0.05	0.12	0.03
*F* value	3.92	5.05	2.94
*F* change	3.92	5.40	0.90
Sig *F* change	0.02	<.001	0.53

*Note*: Standardized beta coefficients are presented. Bold values in parentheses represent *p* values, only the significant values are presented.

^a^ Use of mobility devices = walking aids and wheelchair.

^b^ Location of stroke was transformed to dummy variables with left‐side stroke as the reference category.

^c^ Other = stroke localized in the cerebellum, brainstem, both hemispheres, multifocal stroke, or unidentified.

### Characteristics of participants below the WHO recommended level of PA

The characteristics of the participants who were below the WHO recommended level of PA are presented in Table 3. There were significant differences between those below and above the recommended level of PA for the following variables: age (*p* = .03), marital status (*p* = .03), need for home help (*p* = .03), and use of mobility devices (*p* = .004).

**TABLE 3:** Characteristics of the 106 participants below and 54 participants above the WHO recommended level of physical activity (PA).

	PA <150 min/week	PA >150 min/week	*p* value
Gender			.51
Men	55 (52)	31 (57)	
Women	51 (48)	23 (43)	
Age (y)	74 ± 11; 75, 30–91	71 ± 10; 72, 47–90	**.03**
Marital status			**.03**
Living alone	46 (43)	14 (26)	
Cohabitant	60 (57)	40 (74)	
Vocational situation			.31
Working	15 (14)	11 (20)	
Not working	91 (86)	43 (80)	
Need for home help			**.03**
Independent	84 (79)	50 (93)	
Home help	22 (21)	4 (7)	
Use of mobility devices			**.004**
Walking independently	67 (63)	46 (85)	
Need of mobility devices^a^	39 (37)	8 (15)	
Time since stroke (mo)	35 ± 11; 34, 18–60	36 ± 11; 34, 18–61	.57
First time stroke	87 (82)	49 (91)	.15
Type of stroke			.97
Ischemia	92 (87)	47 (87)	
Intracerebral hemorrhage	14 (13)	7 (13)	
Location of stroke			.53
Right hemisphere	34 (32)	19 (35)	
Left hemisphere	42 (40)	17 (32)	
Cerebellum	7 (6)	6 (11)	
Brainstem	1 (1)	2 (4)	
Other^b^	22 (21)	10 (18)	
Stroke treatment			.25
Thrombolysis/Thrombectomy	21 (20)	15 (28)	
No treatment	85 (80)	39 (72)	

*Note*: Characteristics were analyzed with the *χ* ^2^ test or Mann–Whitney *U* test. Data are presented as *n* (%) and mean ± SD; median, minimum‐maximum.

^a^ Need of mobility devices = walking aids and wheelchair.

^b^ Other = stroke localized in both hemispheres, multifocal stroke, or unidentified.

## DISCUSSION

Increased knowledge of PA >1 year after stroke and its determinants may help us identify those at risk of not meeting the WHO recommendations of PA. The main findings in this study were that 66.3% of the participants were below the WHO recommendations and, thereby, did not meet the WHO recommended level of PA. From the hierarchical regression analysis, the final model explained 13% of the variance in PA with home help as a single significant contributor. Those who did not meet the WHO recommended level of PA were significantly older, more likely to live alone, and in need of home help and mobility devices.

### Self‐reported level of PA

The participants in the present study were less physically active compared to the general adult Swedish population, where 33% report themselves not meeting the WHO recommendations. Among the adult population in the northernmost part of Sweden, the proportion is similar (35%).^26^ Thus, our data confirm previous studies showing that the stroke population is less physically active than healthy controls^11^, ^12^ and do not meet the WHO recommendations of PA to the same extent.

The participants in the present study were similarly or less physically active compared to participants in other stroke studies. Apriliyasari et al.^27^ showed that 63% of people at some point after stroke reported an insufficient level of PA according to the WHO recommendations. In another study, 45% of people 6 months to 5 years after stroke reported an insufficient level of PA.^28^ In addition, 59% of people with a mean time after stroke of 11 months did not meet the WHO recommendations.^15^ These studies were conducted in Indonesia, England, and Benin. As environmental exposures may affect PA after stroke,^29^ cultural and contextual factors such as the outdoor environment, accessibility and distance to rehabilitation facilities, and financial considerations could explain the differences between the populations. The use of various assessment tools may also have contributed to the differences.^30^ Despite geographical location, different assessment tools, and time after stroke, it is clear that many survivors of stroke do not meet the WHO recommended level of PA.

About a third (35.6%) of the participants in the present study were just below the WHO recommendations, with a score of 8–10. It is possible that a small increase in their level of PA could mean that they meet the WHO recommendations. The benefits of PA are substantial and affect residual impairments,^31^ increase recovery,^8^ and reduce the risk of recurrent stroke.^9^, ^32^ Moreover, an increased recovery might stimulate the person to be more physically active and thereby initiate a positive approach to PA. As 20% of stroke cases are recurrences^33^ and the second onset often leads to a worse outcome,^34^ it is important to stimulate PA aiming towards the WHO recommendations.

### Association between the level of PA, sociodemographics, and stroke characteristics

In the hierarchical regression analysis, the final model explained 13% of the variance in PA, with the need for home help as a single significant contributor. A need for home help could imply that the person is older and more affected by the stroke and therefore less likely to participate in PA. This result is in agreement with studies where a more severe stroke, functional dependency, and higher age were associated with physical inactivity 1 year after stroke^35^, ^36^ and where stroke‐related disability was associated with insufficient PA at least 6 months after stroke.^28^ Also, factors that can indicate a positive recovery, such as better walking ability and better balance, have been shown to be associated with a higher level of PA.^11^

Gender was not a significant contributor to the variance in PA, which is in line with previous studies.^15^, ^16^ Only 2 of the 21 studies included in a systematic review showed a significant association between gender and PA.^14^ In addition, there were no significant associations in the present study between the level of PA and the remaining sociodemographic factors, and the included stroke characteristics had no significant contribution to the variance in PA. Few studies have assessed these factors, yet the findings are consistent with some previous studies showing no significant associations between time since stroke, type or location of stroke, and the level of PA.^14^, ^15^ Also, a longitudinal study of the level of PA showed no change over a 12‐month period.^37^

Taken together, these results suggest that the variability in the level of PA after stroke is affected mainly by the person's degree of disability, regardless of other sociodemographics, time since stroke, and current stroke characteristics. As the explanatory power is relatively low, this implies that the variance in PA is mainly explained by other factors not examined in the present study.

### Characteristics of participants below the WHO recommended level of PA

This is, to the best of our knowledge, one of the first studies that have determined the characteristics of survivors of stroke who are below the WHO recommended level of PA. Those of older age and those who needed home help and mobility devices were more likely to be below the WHO recommended level of PA. This is not unexpected, as all these factors could be associated with the degree of disability after stroke and thereby the level of PA. For example, the use of mobility devices has been shown to be significantly associated with the percentage of total sedentary time.^17^ Also, a higher age has been shown to be significantly associated with physical inactivity.^14^ Living alone was also a factor for being below the WHO recommended level of PA in the present study. This result differs from a study conducted in Benin where living alone was instead significantly associated with moderate to intense PA.^15^ A study of older adults (>50 years) in Canada reported that being cohabitant was associated with higher levels of PA.^38^ Being cohabitant could have a positive impact on the level of PA, for example, a motivating partner who assists with transportation. Future studies should therefore explore the importance of the social environment for conducting PA after stroke.

### Methodological considerations

Using a self‐assessment questionnaire specifically developed based on the WHO recommendations of PA enabled us to determine factors of importance for not meeting these recommendations. Assessing PA is complex as it comprises several domains: intensity, frequency, and duration. A recommendation is to include time spent in moderate to vigorous PA,^30^ which is in line with the BHW PA questionnaire. However, self‐assessment tools may overestimate the total amount of PA as well as the intensity of PA, and the possibility of recall bias related to PA questions is well known. A monitoring device can provide more detailed information about the duration and intensity of PA during a day,^39^ although it requires more resources. Also, many studies assess the PA level by recording the number steps per day; this does not, however, cover the intensity of PA and can therefore make it difficult to compare with the WHO recommendations.

### Clinical implications and future research

Our results imply that it is the consequences of the stroke that mainly influence the level of PA and not the stroke characteristics. As we can influence the consequences of stroke through rehabilitation, encouraging PA after stroke is crucial in order to stimulate brain plasticity and recovery.^40^, ^41^, ^42^ As survivors of stroke are capable of behavior change and become physically active even with a history of inactivity,^43^ rehabilitation professionals should focus on how to increase the PA level soon after stroke onset and, in particular, target those of older age, those who live alone, and those who are less physically recovered (ie, need for home help and mobility devices). Several studies have examined stroke‐related factors and their association to PA, as well as facilitators and barriers to conduct PA after stroke.^11^, ^44^, ^45^, ^46^ Yet, few studies relate these findings to the WHO recommendations. Future studies should therefore explore other factors that can determine those at risk of not meeting the WHO recommendations of PA, such as nonmotor impairments (eg, fatigue and mental health), factors in the physical and social environment as well as how to increase the level of PA after stroke.

### Strengths and limitations

The strengths of the present study are the wide range of time since stroke onset and the use of a dataset with very few missing data.^20^ Also, the participants are representative of the global stroke population in terms of the distribution of the type of stroke^47^ and the age of the study participants.^48^ Also, the degree of recovery (presented in the LASINS study)^20^ is similar to recovery patterns described in other studies.^49^ There were also some limitations. The cross‐sectional design does not allow us to determine any causal inferences, so follow‐up data are required. As the study population can be considered to be fairly well recovered (ie, only 29% needed mobility devices and only 16% needed home help), the results should be limited to those with similar disability.

## CONCLUSIONS

Our findings show that a majority of survivors of stroke are physically inactive and do not meet the WHO recommended level of PA. In particular, those of older age, those who live alone, and those who are less physically recovered from their stroke seem to be more likely to report a lower level of PA regardless of gender, vocational situation, time since stroke onset, or current stroke characteristics. Future studies need to assess how other factors characterize those who do not meet the WHO recommendations. This knowledge could help rehabilitation professionals to target interventions and self‐management programs to promote PA among survivors of stroke.

## FUNDING INFORMATION

The study received financial support from the Promobilia Foundation and Strokeforskning Norrland. Maria Kähler is supported by a PhD‐grant from Norrbotten County Council (Region Norrbotten).

## DISCLOSURE

The authors report no conflicts of interest.

## APPENDIX 1

The Swedish National Board of Health and Welfare Physical Activity (BHW PA) questionnaire.^23^, ^24^

1. In a typical week, how much time do you spend on physical exercise that makes you feel out of breath, such as running, gym, or ball sports?

- 0 min/no time
- Less than 30 min
- 30–60 min (0.5–1 h)
- 60–90 min (1–1.5 h)
- 90–120 mins (1.5–2 h)
- More than 120 min (2 h).

2. In a typical week, how much time do you spend on everyday exercise, such as walking, cycling, or gardening? Add up all the time (at least 10 min at a time).

- 0 min/no time
- Less than 30 min
- 30–60 min (0.5–1 h)
- 60–90 min (1–1.5 h)
- 90–150 min (1.5–2.5 h)
- 150–300 min (2.5–5 h)
- More than 300 min (5 h).
